# Integrating Circulating Tumor DNA into Clinical Management of Colorectal Cancer: Practical Implications and Therapeutic Challenges

**DOI:** 10.3390/cancers17152520

**Published:** 2025-07-30

**Authors:** Nikhil Vojjala, Viktoriya Gibatova, Raj N. Shah, Sakshi Singal, Rishab Prabhu, Geetha Krishnamoorthy, Karen Riggins, Nagaishwarya Moka

**Affiliations:** 1 Department of Internal Medicine, Trinity Health Oakland Hospital, Pontiac, MI 48341, USAgeetha.krishnamoorthy@trinity-health.org (G.K.); 2 School of Medicine, Ross University, Miami, FL 33130, USA; 3 Department of Internal Medicine, Kansas University School of Medicine-Wichita, Wichita, KS 66160, USA; rshah3@kumc.edu; 4 Department of Oncology, East Tennessee State University, Johnson City, TN 37614, USA; 5 Department of Hematology Oncology, Dan L Duncan Comprehensive Cancer Center, Baylor College of Medicine, Houston, TX 77030, USA; 6 Department of Hematology Oncology, Lincoln Memorial University, Harrogate, TN 38105, USA

**Keywords:** ctDNA, colorectal cancer, prognosis, surveillance

## Abstract

The American Cancer Society estimates that over 152,000 new cases of colorectal cancer (CRC) were diagnosed in 2024, with more than 105,000 cases affecting the colon and 46,000 involving the rectum. CRC remains the second leading cause of cancer-related deaths in the United States, with an estimated 53,010 deaths in 2024. In the era of precision medicine, which incorporates molecular and environmental information into clinical decision-making, identifying patients harboring a deficiency in Deoxyribonucleic acid (DNA) repair allowed for targeted immunotherapies and significantly reduced CRC-related mortality. A significant advancement in this domain is the application of liquid biopsy, which has emerged as a promising tool for prognostication, guiding therapy, and monitoring treatment response in CRC. This review aims to comprehensively explore the role of liquid biopsy in colorectal malignancies, describing its practical applications, prognostic significance, and potential to revolutionize CRC management in the future. At the end, we also aim to show a schematic representation of showing integration of Circulating Tumor (Ct) DNA in routine clinical management of CRC. The highlight of this article is the structured and evidence-based schematic framework and its integration into future practice. The schematic pathway is designed to optimize ctDNA utilization across various stages of colorectal cancer management.

## Simple Summary

In 2024, over 152,000 people in the U.S. were diagnosed with colorectal cancer, which affects the colon or rectum. It is expected to remain the second-deadliest cancer in the country, with more than 53,000 expected deaths. With new advances in precision medicine, doctors can now find specific problems in a person’s DNA to offer targeted treatments, which has helped lower the death rate. One important new tool is the liquid biopsy—a simple blood test that can help doctors predict how the cancer will behave, choose the best treatment, and track how well it is working. This article reviews how liquid biopsies can be used in treating colorectal cancer and includes a helpful diagram showing how a specific type of test called ctDNA can fit into everyday care. The goal is to make cancer treatment more personalized and effective.

## 1. Introduction

The American Cancer Society estimates that over 152,000 new cases of colorectal cancer (CRC) were diagnosed in 2024, with more than 105,000 cases affecting the colon and 46,000 involving the rectum. CRC remains the second leading cause of cancer-related deaths in the United States, with an estimated 53,010 deaths in 2024 [1]. Despite the morbidity, widespread implementation of secondary prevention strategies and advancements in screening measures have contributed to a decline in overall CRC incidence across both sexes, and longitudinal trends in mortality have been in a plateau phase between 1930 and 2021 [2,3]. The incidence of early-onset CRC, defined as CRC diagnosed in adults younger than 50 years, however, continues to rise, emphasizing the urgent need for new diagnostic methods to enhance early detection and improve overall patient survival and clinical outcomes [2].

In the era of precision medicine, which incorporates molecular and environmental information into clinical decision-making, identifying patients harboring a deficiency in DNA repair allowed for targeted immunotherapies and significantly reduced CRC-related mortality [4,5]. Further, enhanced surveillance techniques with high sensitivity, specificity, and precision have also contributed to improved survival outcomes [6]. A significant advancement in this domain is the application of liquid biopsy, which has emerged as a promising tool for prognostication, guiding therapy, and monitoring treatment response in CRC [7,8]. Circulating tumor cells (CTCs) are tumor-derived cells that have separated and shed into the bloodstream. These CTCs release small DNA fragments into circulation, also known as cell-free DNA (cfDNA). An important fragment of this DNA that contains tumor genetic mutations is more specifically known as circulating tumor DNA (ctDNA). Modern techniques allow for the detection of this small DNA to identify cancer presence and guide future management. The use of CTCs was initially explored in breast and non-small cell lung cancers [9,10]. In 2010, the liquid biopsy technique rapidly expanded its clinical applications to include the detection of cfDNA [11,12,13]. By 2013, the U.S. Food and Drug Administration (FDA) had approved the first liquid biopsy test for monitoring patients with metastatic breast, prostate, and colorectal cancers through the CTC enumeration technique [14]. In 2016, the FDA further approved the detection of the Septin 9 gene methylation (EpiProColon assay) as a biomarker for CRC screening [15]. This review aims to comprehensively explore the role of liquid biopsy in colorectal malignancies, describing its practical applications, prognostic significance, and potential to revolutionize CRC management in the future. At the end, we also aim to show a schematic representation showing the integration of ctDNA in routine clinical management of CRC.

## 2. Methodology

A comprehensive literature search was conducted across multiple databases, including PubMed, Medline, Embase, and Google Scholar. The search strategy employed the following query: ((colon) OR (rectal) OR (colorectal)) AND ((ctDNA) OR (biomarker) OR (cfDNA) OR (DNA)) AND ((cancer) OR (malignancy) OR (carcinoma)). The timeframe for inclusion was restricted to publications from 1 January 2018, to 30 June 2025, to ensure the inclusion of the most recent advancements in colorectal cancer research. All the articles that are published in English only were included.

## 3. Evolution and Current Application of ctDNA in CRC

### 3.1. Historical Aspects of ctDNA

The initial clinical applications of ctDNA emerged from research on CTCs, which were first explored for early breast cancer detection before their applications extended to other malignancies [10,13,16,17,18,19,20,21] CTCs can be isolated based on their distinct biochemical properties or unique surface markers that separate them from normal blood cells [22]. Over time, the extent of circulating tumor-derived biomarker research has significantly broadened to include cell-free microRNAs (cfmiRNAs), mRNAs, extracellular vesicles (EVs), tumor-educated platelets, cell-free proteins, as well as cfDNA [23,24,25,26]. These liquid biopsy markers offer promising results and are widely explored. Cell-free miRNAs and mRNAs are non-coding RNAs that are released by tumor cells into the circulation. Aberrant miRNA or mRNA expression can be used as an indicator for malignancy [27]. Extracellular vesicles, such as exosomes, are involved in intercellular communication, shuttling mRNA, miRNA, proteins, lipids, and DNA that can also be detected during cancer development [28]. Additionally, the interaction between tumor cells and blood platelets has been found to alter platelet RNA expression, resulting in “tumor-educated” platelets, providing insight into tumor evolution and spread [29]. While histopathological evaluation remains the gold standard for cancer diagnosis and treatment decision-making, liquid biopsy offers a minimally invasive measure for disease monitoring and treatment assessment, providing personalized treatment planning [30]. Its multifaceted utility in diagnostic, predictive, and prognostic cancer management makes it a promising and valuable tool in cancer research.

**Figure 1:**
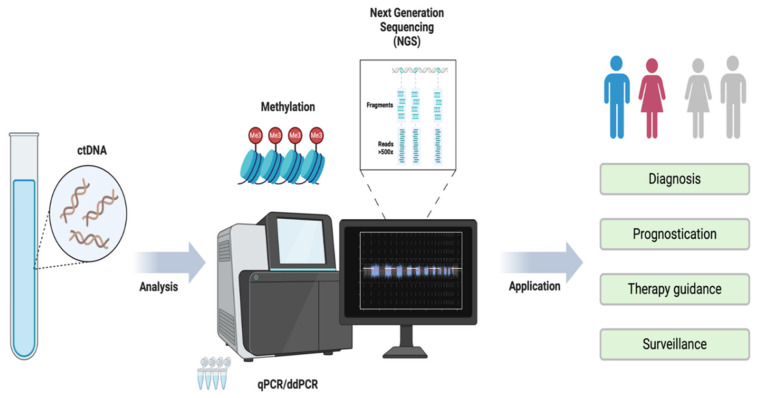
Detection and analysis of ctDNA for application in colorectal cancer management. This figure shows a schematic representation of various methods available for ctDNA detection, which will further guide in diagnosis, prognosis, therapy guidance, and surveillance. ctDNA: Circulating tumor Deoxyribonucleic acids, qPCR: Quantitative polymerase chain reaction, ddPCR: Digital droplet PCR, NGS: Next Generation Sequencing.

ctDNA only contains tumor-derived DNA fragments found in the bloodstream, as opposed to cfDNA, which can be derived from diseased or normal cells. It allows genomic tumor characterization without the need for direct CTC isolation [31]. Different techniques have been developed for ctDNA detection, including quantitative polymerase chain reaction (qPCR), digital droplet PCR (ddPCR), and next-generation sequencing (NGS) [Figure 1]. These methods can be broadly categorized into two major approaches. The first method, tumor-agnostic, employs a preselected mutation panel across all patients, facilitating rapid sequencing but limiting personalization [32]. Each tumor analysis involves the identification of designated, previously recognized mutations regardless of their type and origin. On the other hand, the second method, tumor-informed, uses tumor tissue to create a personalized assay that allows for analysis of patient-specific mutations, enhancing overall sensitivity but with high turnaround time [33,34]. In addition to identifying tumor genetic abnormalities, ctDNA analysis also enables the detection of epigenetic changes, including DNA methylation, for which several assays have been developed over recent years [35,36,37]. Additionally, fragmentomics, which examines the structural and sequential characteristics of plasma cfDNA, has increased the application of liquid biopsy in oncology [38]. The standard PCR-based methods of ctDNA analysis include ddPCR and NGS coupled approaches [30,39]. Digital PCR analysis alone can achieve a high sensitivity and specificity of 98.15% and 88.66%, respectively [40]. While NGS has demonstrated to achieve specificity of up to 99.9% with sensitivity ranging from 38 to 89%, variable based on the driver gene examined [41,42]. Several robust platforms have been created to detect single-nucleotide mutations or whole-genome sequencing (WGS) to assess copy number alteration [43,44,45]. The strengths and weaknesses of these methodologies have been recently reviewed, highlighting the high information throughput of NGS and extensive, comprehensive analysis of WGS [46]. Detection and monitoring of cancer development and prognosis using ctDNA is a key driver for ongoing research and technological advancements in this field. For this reason, liquid biopsy has gathered increased attention, particularly in the management of CRC [47,48,49].

### 3.2. Role of ctDNA in Colorectal Cancer

The clinical application of ctDNA in CRC was first recognized in 2014. A study by Bette Gowda et al. detected ctDNA across multiple malignancies, including CRC, highlighting its potential as a biomarker for tumor burden assessment and treatment response monitoring [50]. Another important study by Diaz et al. further established ctDNA as a clinically relevant tool by demonstrating its ability to identify key mutations (e.g., RAS mutations) and predict sequential therapeutic response [51]. Since then, multiple large Randomized Controlled Trials (RCTs), prospective studies have assessed the efficacy of ctDNA in CRC, particularly for recurrence, survival prediction, and treatment stratification. A specific study by Tie et al. assessed 2-year disease-free survival (DFS) in patients with CRC by comparing a ctDNA-guided strategy to standard clinicopathological criteria. Their findings discovered comparable recurrence results (93.2% vs. 92.4%), further supporting ctDNA as a highly specific potential biomarker for future applications [52,53,54,55].

New results published by Nakamura et al., GALAXY arm of the CIRCULATE-Japan trial, demonstrated the association of molecular residual disease (MRD) detected by ctDNA with recurrence risk from ACT in patients with resectable stage II-IV CRC [56]. The study enrolled over 2000 patients with a median follow-up of 23 months. Recurrence was seen in 78% of MRD-positive patients compared to only 13% in those with negative ctDNA results. Additionally, at 36-month follow-up, DFS was only 16% in patients with ctDNA positivity versus 83% in the ctDNA-negative cohort. Therefore, ctDNA positivity was found to be the single major prognostic factor associated with lower DFS. Moreover, ctDNA positivity was strongly associated with higher mortality in patients with confirmed radiological recurrence, irrespective of its location, and with fewer chances for curative resection in the future. This updated analysis further validates the role of ctDNA in MRD detection and management [56].

Another recent study by Henriksen et al. evaluated recurrence detection using ctDNA analysis in stage II and III CRC patients undergoing curative-intent resection [57]. In the cohort of 839 patients, 131 (15%) experienced recurrence within 12 months after surgery. Further statistical analysis disclosed a robust ctDNA specificity of 98%, but with limited sensitivity of 35%, yielding a negative predictive value (NPV) of 89% and a positive predictive value (PPV) of 75%. CtDNA status after surgical intervention was found to be the most prognostic factor of DFS. The authors also examined recurrence monitoring using serial ctDNA sampling. The cumulative detection was 73% at 12 months after resection and 87% at 31 months. This study showcases the strong prognostic ability of ctDNA in the detection of disease recurrence postoperatively [57]. In a systematic review and meta-analysis led by Nassar et al. that analyzed a cohort of 1022 patients with locally advanced rectal cancer, positive ctDNA was associated with a significantly increased risk of distant metastasis [58]. Patients with preoperative ctDNA positivity had a fivefold increased risk of developing distant metastasis, while those with positive ctDNA postoperatively had more than six times the risk. Additionally, patients with overall ctDNA positivity, regardless of surgical status, had more than 12 times the risk of disease relapse after treatment [58]. These findings could be used to identify patients with increased risk of metastasis and relapses, providing potential for improved therapy management (Table 1 and Table 2).

### 3.3. Clinical Implications and Challenges of Liquid Biopsies in the Management of Colorectal Cancer

**Figure 2:**
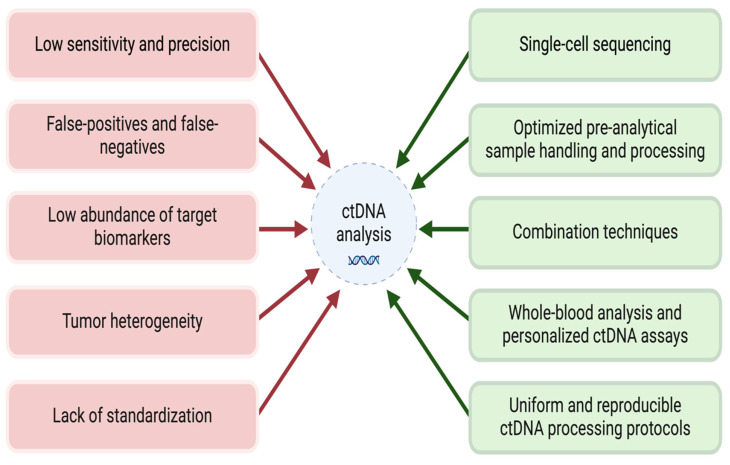
Current challenges of ctDNA analysis and proposed practical solutions. This is a flowchart depicting the challenges of the applications of ctDNA and practical solutions.

Despite the significant promise of ctDNA as a non-invasive biomarker in CRC management, its clinical application is limited by several challenges in sensitivity, specificity, false-positive and false-negative results, tumor-derived ctDNA abundance, heterogeneity of tumors, and lack of standardization. In this review, we further carefully analyze these challenges and propose potential strategies to overcome them [Figure 2]. Addressing these issues is essential to improve the reliability of ctDNA for early cancer detection, MRD monitoring, and therapeutic response assessment, allowing for its integration into clinical practice.

- Screening and Diagnostic Evaluation
  While liquid biopsies offer a minimally invasive alternative to traditional tissue biopsies, they generally exhibit lower sensitivity and precision. The detection sensitivity is often limited by the low levels of ctDNA, particularly in early stages of CRC. This can result in false negatives, where ctDNA is not detected despite the presence of residual disease. Dilution of ctDNA by the presence of DNA from healthy non-malignant cells can also lead to inaccurate detection of cancer-specific mutations, generating aberrant results. One strategy to address this limitation is single-cell analysis, which allows detection of CRC mutations using single-cell sequencing that might be missed by bulk sequencing methods. This enhances both the sensitivity and specificity of ctDNA assays, leading to more accurate detection of MRD and early recurrence [81,82]. Using tumor-informed methods to identify patient-specific mutations can also improve ctDNA detection sensitivity, facilitating more exact mutation identification. Standardizing plasma separation and storage protocols to minimize degradation of ctDNA before its analysis can prevent false-positive and false-negative results. Additionally, implementing advanced processing techniques, such as specialized blood collection tubes and double plasma centrifugation, can further improve assay reliability. However, these methodological changes must be carefully weighed against processing complexity and its associated cost for feasibility in routine clinical practice [83,84].
- Low Amount of Target Biomarkers
  ctDNA is released into the bloodstream primarily through apoptosis, necrosis, or secretion from tumor cells. However, in early stages, the tumor burden is minimal, shedding only very small amounts of DNA into circulation. This poses a challenge for the detection of ctDNA in early cancer management [50]. Additionally, with a half-life of only 20–60 min, ctDNA is rapidly removed from circulation, limiting the detection ability even further. This subsequently necessitates highly sensitive analytical methods and techniques [85]. While methylation-based approaches have enhanced ctDNA detection rates, their sensitivity remains low to achieve acceptable detection when used in isolation [86]. To address this, an approach utilizing combination techniques may be required. The integration of genetic and epigenetic alterations, such as combining hotspot mutations with the Screening for the Presence of Tumor by Methylation and Size (SPOT-MAS) assay, developed by Nguyen et al., into a multimodal tool that has previously demonstrated significant improvement in the detection of target biomarkers [87].
- Tumor Heterogeneity
  Tumors exhibit considerable genetic variations across different genetic regions, making it challenging to capture a comprehensive molecular profile of their heterogeneity. Studies indicate that not all tumor mutations are detectable in plasma due to limited shedding and tumor heterogeneity, which can significantly compromise the accuracy of liquid biopsy analyses [88,89]. This limitation affects treatment decisions, as tumors with variations in genetic markers may respond differently to targeted therapies. To overcome this, incorporating additional quality control measures, such as paired whole-blood analysis, can help distinguish tumor-derived mutations from clonal hematopoiesis of indeterminate potential (CHIP) and improve ctDNA assay results [90]. Furthermore, designing personalized ctDNA assays tailored to individual tumor mutation profiles can enhance sensitivity and specificity, allowing superior mutation detection. Hybrid-capture ctDNA sequencing utilizing customized target-enrichment panels has demonstrated high efficacy in detecting MRD, offering a more refined strategy to disease monitoring and treatment optimization [91].
- Lack of Standardization
  The lack of standardized protocols and clinically validated guidelines for ctDNA collection, processing, and analysis can lead to unpredictable results, complicating the implementation of ctDNA assays into clinical practice. Variability in methodologies contributes to inconsistencies and poor reliability of liquid biopsy results. Additionally, the lack of a universally accepted comprehensive ctDNA marker profile imposes an extra layer of complexity for the accurate identification of tumor mutations. Establishing standardized guidelines for variant classification, interpretation, and reporting is necessary to ascertain the clinical utility of ctDNA assays. Collaborative efforts between international societal groups are essential to harmonize the best practices. Differences in blood collection techniques, plasma processing, and DNA extraction methods can significantly impact ctDNA yield and quality, making cross-study comparisons difficult. The American Society of Clinical Oncology (ASCO) and the College of American Pathologists (CAP) have emphasized the need for uniform and reproducible quantification methods to ensure comparable results and interoperability across different laboratories [92,93]. Implementation of internal quality control (IQC) measures and participation in external quality assessment (EQA) programs can improve the reliability and clinical strength of ctDNA assays. Recent data shows that only 45.6% of laboratories engage in EQA programs, underscoring the urgent need for their broader adoption [94].

### 3.4. Current Progress and Applications in the Management of Colorectal Cancer

**Figure 3:**
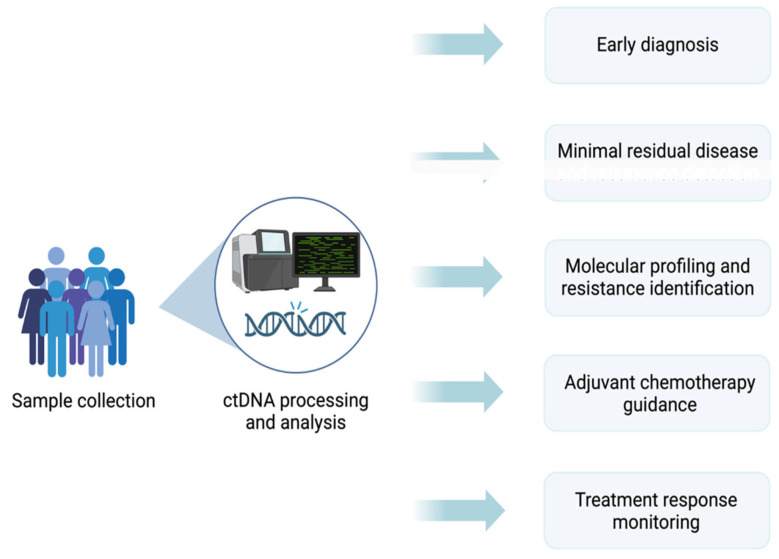
This figure demonstrates the current application of the ctDNA in the management of colorectal cancer, starting from early diagnosis to monitoring treatment response.

ctDNA has demonstrated significant utility in the detection of MRD, post-treatment surveillance for early recurrence, molecular profiling, and prognostication of therapeutic efficacy in CRC [95,96] (Figure 3). Notable ctDNA positivity following curative-intent resection has been identified to be a strong predictor of recurrence. Its monitoring enables early detection of residual disease before radiological evidence of relapse [97]. Compared to standard imaging modalities such as computed tomography (CT), ctDNA analysis enables MRD detection beyond the limits of radiographic imaging [98]. Additionally, ctDNA has been demonstrated to predict the recurrence of CRC by an average of 9.4 months earlier compared to tomography [99]. This role in MRD detection can assist in guidance of adjuvant chemotherapy decisions, potentially minimizing overtreatment in patients with undetectable ctDNA levels while ensuring appropriate intervention for those at higher risk of relapses. Additionally, the ctDNA expression allows for guiding the need for post-surgical intervention. Patients who demonstrate ctDNA negativity may be spared from adjuvant chemotherapy without compromising their end outcomes and reducing the overall exposure to additional side effects. In contrast, patients with detectable ctDNA levels postoperatively may gain significant benefit from chemotherapy, as the presence of ctDNA indicates residual disease and a high risk of recurrence.

ctDNA is also effective in de-escalation of adjuvant chemotherapy without impacting progression-free survival (PFS) [100]. A study investigating ctDNA-guided treatment stratification in CRC showed that patients with decreased in RAS clonal mutations exhibited superior therapeutic response and PFS (HR 0.21; 95% CI 0.06–0.71%; *p* = 0.01) and overall survival (OS) (HR 0.28; 95% CI 0.07–1.04%; *p* = 0.06) [101]. As a result, ctDNA analysis is being increasingly incorporated into routine adjuvant therapy planning for CRC patients, particularly for monitoring of recurrence after surgery [102,103].

In metastatic CRC, ctDNA is gaining an increasing reputation as a tool to monitor chemotherapy response as well as targeted therapy response. Dhiman et al. showed that patients with rising ctDNA had a 90% recurrence rate compared to only 21% in those with stable ctDNA levels, highlighting the utility of post-treatment ctDNA surveillance [71]. This monitoring approach facilitates tracking of tumor burden and identification of molecular resistance mechanisms. Another study by Zhou et al. investigated the potential of ctDNA in predicting response to neoadjuvant chemotherapy (nACT), monitoring tumor burden, and prognostication. Their findings suggest that ctDNA may serve as a real-time indicator of disease status. Multiple additional studies have demonstrated the potential of ctDNA application in the screening of CRC with epigenetic profiling, emerging as a promising biomarker for both early diagnosis and longitudinal disease surveillance [104,105,106]. Furthermore, several multi-cancer early detection tests (MCED) (e.g., Galleri) are undergoing evaluation, though further validation is still required before clinical implementation [107].

The collective data demonstrate that ctDNA-positive patients post curative resection have a mean recurrence of 63.3%, a much higher rate when compared to 19.1% in patients with ctDNA-negative results. This three-fold difference signifies the prognostic power of this tool for monitoring the recurrence risk and potentially guiding further therapy decision-making. However, the ctDNA positive cohort also exhibited a greater variability in recurrence rates (SD ± 23.9%), which may be attributed to differences in timing of ctDNA assessment, treatment regimen, as well as underlying molecular subtypes of CRC. A more standardized and systematic approach is necessary to accurately evaluate the extent of variability within this cohort. Meanwhile, the recurrence rates in the ctDNA-negative group had a more consistent pattern (SD ± 10.3%), suggesting a potentially greater reliability in identifying patients with a favorable clinical outcome [104,107].

This indicates that the patients who demonstrate ctDNA positivity postoperatively are at a markedly increased risk of disease recurrence, justifying a more robust and vigorous cancer surveillance with a low threshold for early intervention. On the other side, patients with ctDNA negative results are at a lower risk of recurrence and are less likely to require adjuvant intervention or may benefit from early therapy de-escalation, avoiding further unnecessary exposure to chemotherapy and its associated toxic effects. These findings demonstrate the potential of effective risk-stratification in early disease, leading to more favorable long-term outcomes.

Finally, several ongoing studies are underway to further validate and expand ctDNA application in CRC management. Notable trials include the CIRCULATE JAPAN VEGA and ALTAIR trials, Dynamic III, MEDOCC-CrEATE, ACT3, and CAP-LR trials, which are assessing the utility in adjuvant therapy decision guidance and post-operative surveillance [108,109,110,111]. These trials aim to determine whether ctDNA-guided risk stratification can surpass conventional clinicopathologic criteria in treatment planning.

### 3.5. Commercially Available ctDNA Tests in CRC

Several commercially available ctDNA assays have been developed for minimal residual disease (MRD) detection and longitudinal monitoring in colorectal cancer (CRC). These assays vary in assay design (tumor-informed vs. tumor-naïve), analytical sensitivity, turnaround time, and cost. Tumor-informed platforms such as Signatera™ (Natera, Austin, TX, USA) and FoundationOne^®^ Tracker (Foundation Medicine, Boston, MA, USA) require prior sequencing of tumor tissue to design personalized panels, allowing for higher sensitivity but limiting feasibility in settings where archival tumor material is not available or sequencing infrastructure is lacking. Tumor-naïve platforms like Guardant Reveal™ bypass the need for tumor tissue by detecting common genomic alterations and methylation signatures, which may offer a more accessible solution in certain contexts, albeit at potentially lower sensitivity. RaDaR™ (Inivata, Cambridge, UK) and Safe-SeqS, an academically developed platform at Johns Hopkins, represent other approaches under active clinical and translational evaluation. While these technologies are reshaping MRD detection in high-income countries, their high cost and logistical complexity limit broader application in low- and middle-income countries (LMICs), where infrastructure for plasma DNA extraction, sequencing, and interpretation is often underdeveloped. The table below summarizes key characteristics of the most widely used ctDNA assays in CRC (Table 3).

### 3.6. Current Schematic Strategy and Future Direction

The future of CRC management is centered around the widespread adoption of ctDNA as a foundation of personalized oncology. The integration of ctDNA into clinical practice is revolutionizing the diagnosis and management of cancers, particularly in MRD and adjuvant therapy guidance. While its clinical utility continues to grow, additional ongoing research remains vital to standardize its application and integration into today’s clinical practice. Given its rapid development and significant clinical relevance, ctDNA is on track to become a standard tool in guiding CRC management. Although RCTs have demonstrated variability in result reproducibility in studies with ctDNA compared to conventional therapeutic strategies, ctDNA is still very promising for disease surveillance and is a meaningful adjunct to imaging modalities. Emerging advancements in ctDNA detection technologies, including fragmentomics and multi-omics integrations, are projected to improve assay sensitivity and specificity, further refining tumor assay approaches for MRD detection and disease surveillance [110]. Ultimately, the evolution of liquid biopsy cancer management using ctDNA has the potential to eliminate our heavy reliance on traditional invasive tissue biopsy and repeated imaging, allowing for precision cancer care.

**Figure 4:**
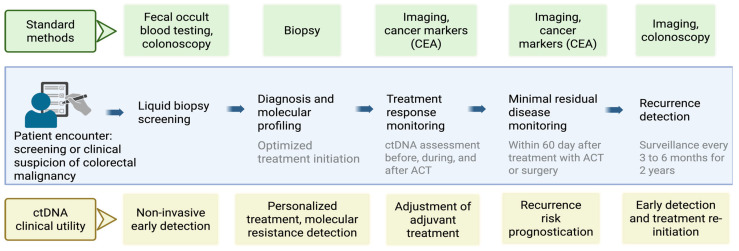
A schematic diagram shows ctDNA application in the management of colorectal cancer. The top green colored flow represents current standards of screening, diagnosis and follow-up. The bottom flow chart represents the futuristic applications of ctDNA at various stages in the management of CRC.

Building on our current literature review and latest available evidence regarding the clinical application of ctDNA, we propose a structured and evidence-based schematic framework for its integration into future practice. This pathway is designed to optimize ctDNA utilization across various stages of cancer management. Figure 4 presents a proposed clinical workflow integrating ctDNA into the management of CRC, aiming to enhance early detection, guide treatment decisions, and improve recurrence surveillance. Traditionally, CRC screening, diagnosis, and follow-up rely on conventional screening methods (like fecal occult blood testing, colonoscopy), biopsy, and tumor markers and imaging during follow-up. The proposed algorithm introduces ctDNA at multiple stages, beginning with non-invasive liquid biopsy screening at the time of clinical suspicion. Following diagnosis, ctDNA facilitates molecular profiling to tailor treatment and detect resistance mechanisms. During and after adjuvant chemotherapy, serial ctDNA assessments provide a dynamic view of treatment response, enabling early adjustments. For MRD, ctDNA testing within 60 days post-treatment is used to detect molecular relapses before clinical recurrence. Subsequently, in the surveillance phase, ctDNA complements imaging by enabling recurrence detection at earlier timepoints, with monitoring suggested every 3–6 months for two years. This approach leverages the high sensitivity of ctDNA for molecular residual disease, supporting the timely re-initiation of therapy. Overall, the algorithm repositions ctDNA as a central tool for personalized CRC care, spanning early detection, treatment guidance, and recurrence monitoring, especially valuable in advancing precision oncology. However, before the implementation of the proposed strategy, some of the practical challenges need to be addressed, especially when the universal application of this is expected.

Despite encouraging data from key studies, significant discrepancies exist across clinical trials in assay platforms, timing of testing, and clinical endpoints, posing challenges for standardization and equitable implementation, especially in low- and middle-income countries (LMICs).

One of the most cited studies, the DYNAMIC trial, demonstrated that a ctDNA-guided approach in stage II CRC reduced adjuvant chemotherapy use without RFS [68]. Yet, other trials such as COBRA (NCT04068103) and CIRCULATE-Japan (GALAXY study) differ in design. COBRA is a phase 2 study and was stopped prematurely because it failed to meet the primary endpoint [112]. DYNAMIC used tumor-informed bespoke assays, while others have used fixed panels or tumor-naïve approaches. These differences influence sensitivity, turnaround time, and feasibility, particularly in LMIC settings where infrastructure for high-throughput NGS may be lacking. Moreover, the definition of “ctDNA positivity” varies, and the optimal timing for post-surgical blood draws (e.g., 4 weeks vs. 8 weeks) is not standardized, complicating comparisons across trials [59,65].

In LMICs, systemic limitations exacerbate these challenges. Most public hospitals lack the infrastructure for tumor sequencing and longitudinal plasma sampling, limiting the feasibility of tumor-informed ctDNA assays. Cold chain logistics, PCR/NGS capability, and standardized bioinformatics support are often confined to academic or private centers [116]. Even where testing is theoretically available, the cost of commercial assays, ranging from $1000–$3000 per test, is a major barrier, often unreimbursed by public health systems [117].

Moreover, most LMICs do not have regulatory guidelines incorporating ctDNA into CRC care algorithms. Without structured policy frameworks, ctDNA testing risks fragmented and potentially inappropriate use, particularly in private settings where patient awareness may outpace physician comfort with interpretation. Additionally, ctDNA assays validated in Western populations may not account for region-specific biology or germline variants, underscoring the need for local validation [118].

## 4. Conclusions

In conclusion, while ctDNA offers a transformative pathway toward individualized therapy in CRC, particularly in the setting of MRD, its implementation is hindered by significant barriers. Harmonization of clinical trial designs, subsidization of ctDNA technologies, expansion of molecular diagnostic infrastructure, and the generation of locally relevant data are essential steps toward equitable integration of ctDNA into global CRC care.

## Figures and Tables

**Table 1:** Summary of randomized controlled trials and prospective studies assessing the application of ctDNA in colorectal cancer.

Author	Year	Design	Timing of ctDNA Assessment	Primary Endpoint	Findings
Reinert et al. [59]	2019	Multicenter prospective study	Before surgery, 30 days after, and every third month for 3 years	Recurrence rates	70% recurrence in ctDNA-positive patients (95% CI, 34.2–93.1%); recurrence in the ctDNA negative group was 11.9% (95% CI, 6.3–20.1%)
Tarazona et al. [60]	2019	Prospective cohort study	6–8 weeks after surgery, and every 4 months for up to 5 years	DFS	57.1% recurrence in the ctDNA-positive after surgery and 85% after ACT
Tie et al. [61]	2019	Multicenter cohort study	4–10 weeks after surgery and after ACT	3-year RFI	ctDNA-positive patients had RFI of 30% (95% CI, 9–55%) at 3 years, and ctDNA-negative patients had 77% (95% CI, 60–87%)
Tie et al. [62]	2020	Multicenter cohort study	4–6, 6–8, and 8–10 weeks after surgery	RFS	RFS was inferior for the post-surgery ctDNA-positive group at 5 years (38.6% vs. 85.5%; HR, 7.56; 95% CI, 4.85–11.79%)
Zhou et al. [63]	2021	Multicenter prospective study	Before nCRT, one cycle after nCT, 7 weeks after nCRT, before surgery, and within 1 month after	Metastasis-free survival	ctDNA is an independent predictor of MFS (HR, 1.267; *p* < 0.001)
Henriksen et al. [64]	2021	Prospective cohort study	2–4 weeks after surgery, before initiation of ACT	RFS	18% recurrence rate in ctDNA negative group, 80% in ctDNA positive group
Taieb et al. [65]	2021	Randomized controlled trial	6–8 weeks after surgery	DFS and OS	3-year DFS rate 66.39% in ctDNA positive and 76.71% for ctDNA negative group (*p* = 0.015)
Parikh et al. [66]	2021	Prospective study	Before surgery, 4 weeks after surgery, 4 weeks after ACT	Detection of ctDNA and RFS	100% recurrence in the CtDNA-positive group and 24.5% recurrence in the CtDNA-negative group
Liu et al. [52]	2022	Multicenter randomized trial	During and after NAT and before TME	RFS at 3 years	ctDNA predictor of recurrence in high risk (HR = 21.27; 95% CI, 5.15–87.92%); or low risk (HR = 16.39; 95% CI, 1.46–184.30%)
Li et al. [67]	2022	Prospective cohort study	1 week before ACT and 2–4 weeks after ACT	RFS	3-year RFS in the ctDNA-positive group was 45.5% and in the ctDNA negative group was 72.7%; 24.8% recurrence ctDNA negative group and 54.5% recurrence in the ctDNA positive patients after ACT
Tie et al. [68]	2022	Multicenter randomized controlled trial	4 or 7 weeks after surgery	RFS at 2 years	2-year recurrence-free survival was 93.2% in the ctDNA-guided and 92.4% in standard management
Kotani et al. [69]	2023	Multicenter prospective study	4–12 weeks after surgery	DFS	9.5% recurrence in the ctDNA negative group and 61.4% in the ctDNA positive patients
Leonardi et al. [70]	2023	Randomized controlled trial	After surgery and after ACT	Post-surgery false negative cases	34% relapse in the CTDNA-positive group, 9% relapse in the CTDNA-negative group
Dhiman et al. [71]	2023	Prospective cohort study	4–6 weeks after surgery, 4–6 weeks, and every 3 months for 1 year	RFS	90% recurrence in the rising ctDNA levels group vs. 21% in the stable ctDNA group
Lygre et al. [72]	2024	Prospective observational study	1 month after surgery, 3 months, and then every 6 months	RFS	Higher recurrence in ctDNA-positive patients vs. ctDNA-negative (HR: 172.91; 95%CI: 8.70 to 3437.24%)
Morris et al. [73]	2024	Multicenter prospective study	At 6 months after ACT	Clearance of ctDNA, RFS	Clearance of ctDNA was observed at 43% (95% CI 10–82%) in the control arm and 11% (95% CI 0.3–48%) in the experimental arm (*p* = 0.98)
Henriksen et al. [57]	2024	Multicenter prospective study	Within 60 days after the operation and every 3–4 months for up to 36 months	RFS	ctDNA detection was prognostic of recurrence (HR 11.3, 95% CI 7.8–16.4%)
Kasi et al. [74]	2024	Multicenter prospective study	4–12 weeks after surgery	DFS	44.2% with ctDNA positive had recurrence; ctDNA positive had significantly worse DFS compared to ctDNA negative (HR = 124.3, 95% CI: 29.8–518.7%)
Yu Kami et al. [75]	2024	Multicenter prospective study	1, 3, 6, 9, 12, 18, and 24 months post-surgery until recurrence	DFS	ctDNA positive patients were 5 times more likely to recur vs. ctDNA negative patients (HR: 5.4, 95%CI: 3.58–7.67%)
Slater et al. [54]	2024	Multicenter prospective study	Before and after surgery, after ACT, every 3 months for year 1 and every 6 months for 2 years after	RFS at 2 years	RFS in ctDNA positive patients was 50.4% and 91.1% in the ctDNA negative group (95% CI, 84.1–95.1%)
Parikh et al. [53]	2024	Multicenter prospective study	Before surgery, 3 and 10 weeks after surgery, and every 12 to 24 weeks for up to 5 years	3-week ctDNA detection rate, RFS and OS	94.7% recurrence in the ctDNA positive and 43.5% recurrence in the ctDNA negative group; sensitivity (40.8–73.6%), specificity (62.3–99.5%)
Nakamura et al. [56]	2024	Multicenter prospective study	4, 12, 24, 36, 48, 72, and 96 weeks after surgery until recurrence	DFS and OS	78.27% recurrence in the ctDNA positive group and 13.14% in the ctDNA negative group; 24-month OS 83.20% in the ctDNA positive group vs. 99.30% in the ctDNA negative group.

ACT: Adjuvant chemotherapy; DFS: Disease-free survival; RFS: Recurrence-free survival; RFI: Recurrence-free interval; OS: Overall Survival; ctDNA: Circulating tumor DNA; NAT: Neoadjuvant therapy; TME: Total mesorectal excision; nCRT: Neoadjuvant chemoradiotherapy; nCT: Neoadjuvant chemotherapy; HR: Hazard Ratio; CI: Confidence intervals.

**Table 2:** Summary of meta-analyses evaluating the applications of ctDNA in guiding the recurrence of colorectal cancer.nCRT:.

Author	Year	Population	Findings
Jones et al. [76]	2021	2823	Poor OS (HR 2.2, 95% CI 1.79–2.69%) and PFS (HR 3.15, 95% CI 2.10–4.73%) in the ctDNA-positive groups after treatment.
Callesen et al. [77]	2022	6930	High baseline ctDNA is associated with short PFS (HR = 2.2; 95% CI 1.8–2.8%; *n* = 509) and OS (HR = 2.4; 95% CI 1.9–3.1%; *n* = 1336)
Faulkner et al. [78]	2022	3002	Worse PFS with ctDNA positive at the first liquid biopsy post-surgery [HR: 6.92, 95% CI: 4.49–10.64%]
Do et al. [79]	2023	3311	ctDNA positive groups had a higher risk of recurrence vs. the ctDNA negative group (RR = 7.73, 95% CI: 5.73–10.42%)
Min et al. [55]	2023	8076	Combined sensitivity of 0.723, specificity of 0.920, and diagnostic OR 23.30 (95%CI: 9.3–57.9%) with an AUC of 0.860
Chang et al. [80]	2023	475	ctDNA positive after nCRT had worse RFS (HR = 9.16, 95% CI, 5.48–15.32%), worse OS (HR = 8.49, 95% CI, 2.20–32.72%), and worse pCR results (OR = 0.40, 95% CI, 0.18–0.89%)
Nassar et al. [58]	2024	1022	Preoperative ctDNA + 5x risk of distant metastasis (RR [95% CI] 5.03 [3.31–7.65%], *p* < 0.001), postoperative ctDNA + 6x risk of distant metastasis (RR [95% CI] 6.17 [2.38–15.95%], *p* < 0.001)

PFS: Progression-free survival; RFS: relapse-free survival; OS: overall survival; HR: hazard ratio; RR: risk ratio; ctDNA: circulating tumor DNA; nCRT: neoadjuvant chemoradiation therapy; pCR: pathological complete response rates; AUC: area under the curve; OR: odds ratio.

**Table 3:** Commercially available ctDNA tests for CRC management.

Assay Name	Company	Assay Type	Technology	Approximate Cost (USD)	MRD Use	Turnaround Time	Notes
Signatera™	Natera	Tumor-informed, bespoke	NGS-based multiplex PCR	~$3500 per timepoint	Yes	10–14 days	Requires initial tumor tissue for personalized assay design. Widely used in MRD trials (e.g., DYNAMIC, CIRCULATE-US) [68,112]
Guardant Reveal ™ [113]	Guardant Health	Tumor-naïve	Targeted hybrid-capture NGS	~$2000–$2500	Yes	7–10 days	Does not require tumor tissue. Includes methylation and genomic alterations [113].
FoundationOne^®^ Tracker [112]	Foundation Medicine	Tumor-informed	Hybrid-capture NGS	~$3000–$3500	Yes	10–14 days	Requires FFPE tissue; used for longitudinal monitoring [114].
Safe-SeqS	Johns Hopkins/PGDx	Tumor-informed	NGS with molecular barcoding	Research use only	Yes	Variable	Used in academic settings; basis for some platforms [115].

This table depicts the commercially available ctDNA testing in CRC patients. Prices are approximate and vary by geography, insurance coverage, and institutional contracts. In LMIC settings, costs are often entirely out-of-pocket, posing substantial barriers to access. Turnaround times reflect optimal logistics, excluding delays from tissue retrieval or customs (for international shipping). Tumor-informed assays require sequencing of archival tumor tissue, which may not be available or retrievable in all LMIC contexts. PCR: Polymerase chain reaction, NGS: Next generation sequencing, MRD: minimal residual disease.

## Data Availability

Not applicable.
